# Insulin Resistance: A Marker for Fat-to-Lean Body Composition in Japanese Adults

**DOI:** 10.3390/nu15224724

**Published:** 2023-11-08

**Authors:** Masahiro Matsui, Akira Fukuda, Saori Onishi, Kosuke Ushiro, Tomohiro Nishikawa, Akira Asai, Soo Ki Kim, Hiroki Nishikawa

**Affiliations:** 1 Second Department of Internal Medicine, Osaka Medical and Pharmaceutical University, Takatsuki 569-8686, Japan; 2 Health Science Clinic, Osaka Medical and Pharmaceutical University, Takatsuki 569-8686, Japan; 3 Department of Gastroenterology, Kobe Asahi Hospital, Kobe 653-8501, Japan

**Keywords:** insulin resistance, fat, skeletal muscle, fat and muscle balance, Japanese adults

## Abstract

We sought to investigate the relationship between insulin resistance (IR) and body composition as assessed by bioelectrical impedance analysis in Japanese health check-up recipients (1186 men and 1441 women). IR was defined as a Homeostasis Model Assessment of IR (HOMA-IR) ≥ 2.5. In body-composition-related parameters, the fat mass index (F index) was defined as fat mass divided by the height squared (kg/m^2^). The fat-free mass index (FF index) was defined as fat-free mass divided by the height squared (kg/m^2^). The F index to FF index ratio (F-FF ratio) was defined as the F index divided by the FF index. Factors related to HOMA-IR were examined. The median HOMA-IR was 1.54 in men and 1.30 in women (*p* < 0.0001). The median F index was 4.9 kg/m^2^ in men and 6.1 kg/m^2^ in women (*p* < 0.0001). The median FF index was 18.2 kg/m^2^ in men and 15.1 kg/m^2^ in women (*p* < 0.0001). The median F-FF ratio was 0.272 in men and 0.405 in women (*p* < 0.0001). The F-FF ratio was an independent factor associated with HOMA-IR in the multivariate analysis in both genders, while the F index and FF index were not in both genders. In conclusion, fat and skeletal muscle balance can be controlled by IR in Japanese adults.

## 1. Introduction

Skeletal muscle accounts for about 50% of body weight, and at rest, about 15% of the circulating blood volume is supplied to skeletal muscle, and about 20% of the oxygen consumed in the body is consumed by skeletal muscle [1]. Blood glucose is transported to skeletal muscle, where it is metabolized by oxygen. Continued aerobic exercise increases oxygen uptake and improves glucose metabolism in skeletal muscle. Endurance exercise improves aerobic metabolism and can reduce hyperglycemia in diabetic patients [2,3,4]. Insulin resistance (IR) is defined as “a condition in which insulin sensitivity in tissues is reduced and insulin action is inadequate” [5]. In patients with IR, glucose uptake into skeletal muscle and adipose tissue is reduced, and glycogenesis in the liver is uncontrolled [6]. Patients with sarcopenia, who have reduced skeletal muscle mass, are forced to release glucose into the bloodstream due to reduced uptake of glucose into the muscles, and thus, blood glucose levels tend to rise [7]. IR decreases muscle protein anabolism by insulin-like growth factor-1 (IGF-1), etc. [8].

It is a well-known fact that ectopic fat accumulation in the liver and IR are likely to occur in obese individuals, and one of the mechanisms of IR is impaired insulin signaling. Ectopic fat accumulation is thought to be one of the causes of impaired insulin signaling [9]. On the other hand, adiponectin, a good adipocytokine secreted by adipocytes, promotes the burning of ectopic fat in the liver and skeletal muscle. Blood levels of adiponectin are decreased by obesity and may act in a facilitative manner on the accumulation of ectopic fat [10]. However, a lot of aspects of the IR-adipose tissue-skeletal muscle relationship remain unclear in Japanese adults [11]. Solving these clinical research questions appears to be clinically meaningful.

Body composition analyzers can be easily used at home to measure fat mass and lean body mass in a minimally invasive manner. Measurement of skeletal muscle mass by the bioelectrical impedance analysis (BIA) method is currently recommended in the criteria for the diagnosis of sarcopenia [12,13]. The purpose of this study was to investigate the relationship between IR and body composition as assessed by BIA in Japanese health check-up recipients.

## 2. Patients and Methods

### 2.1. Patients and Body Composition Analysis

Between February 2022 and May 2023, a total of 2814 consecutive Japanese subjects with both data for Homeostasis Model Assessment of Insulin Resistance (HOMA-IR) and body composition as evaluated by BIA were found in our medical record. Of these, 140 men and 47 women received antidiabetic therapy, and thus, they were excluded from this analysis to avoid bias. A total of 2627 Japanese subjects were therefore analyzed in our analysis. All study subjects received health check-ups at the Osaka Medical and Pharmaceutical University (OMPU) Health Sciences Clinic (OMPU attached facility). IR was defined as HOMA-IR 2.5 or more [14].

In the OMPU Health Sciences Clinic, TANITA (a body composition analyzer with automatic height meter, DC-270A-N, Tokyo, Japan) has been used as a body composition analyzer, and it is a minimally invasive measuring instrument. Measurements were performed in the resting and standing positions after obtaining consent for body composition measurements from each subject. The subjects were in a fasted state. Total fat-free mass (kg) and total fat mass (kg) were able to be measured, but appendicular fat mass and appendicular fat-free mass were not able to be measured by our body composition analyzer. The fat mass index (F index) was defined as fat mass divided by the square of height (kg/m^2^). The fat-free mass index (FF index) was defined as fat-free mass divided by the square of height (kg/m^2^). The F index to FF index ratio (F-FF ratio) was defined as the F index divided by the FF index. Total body muscle mass and total fat-free mass reportedly correlated extremely well with appendicular skeletal muscle mass [15].

### 2.2. Our Study

First, we examined the relationship between HOMA-IR and body composition-related parameters (i.e., F index, FF index, and F-FF ratio). Next, parameters related to HOMA-IR were examined by univariate and multivariate analysis. We obtained ethical approval for the study from the ethics committee of OPMU hospital (approval no. 2023-111), and the protocol strictly observed all regulations of the Declaration of Helsinki. Subject consent regarding this study was waived due to the retrospective analysis of our study.

## 3. Statistics

In the two-group comparison (continuous parameters), Student’s *t*-test, Mann-Whitney *U*-test, or Pearson’s correlation coefficient *r* were used, as appropriate. Factors with statistical significance for the correlation with HOMA-IR were subjected to multivariate regression analysis with multiple predictive variables using the least squares method to choose candidate variables. Unless otherwise noted, data are shown as a number or median (range) value. We considered variables of *p* < 0.05 as statistically significant. JMP 17.0.0 software (SAS Institute, Cary, NC, USA) was used to perform statistical analyses.

## 4. Results

### 4.1. Baseline Characteristics

Baseline characteristics in this study (*n* = 1186 in men and *n* = 1441 in women) are demonstrated in Table 1. The median (range) age was 67 years (25–89 years) in men and 63 years (20–90 years) in women (*p* < 0.0001). The median (range) body mass index (BMI) was 23.0 kg/m^2^ (14.3–45.7 kg/m^2^) in men and 21.2 kg/m^2^ (13.3–37.5 kg/m^2^) in women (*p* < 0.0001). Fatty liver (FL) on ultrasonography (US) was found in 582 subjects (49.1%) in men and 385 subjects (26.7%) in women (*p* < 0.0001). The median (range) HOMA-IR was 1.54 (0.24–18.45) in men and 1.30 (0.13–8.95) in women (*p* < 0.0001). IR (i.e., HOMA-IR ≥ 2.5) was found in 263 subjects (22.2%) in men and 194 subjects (13.5%) in women.

**Figure 1:**
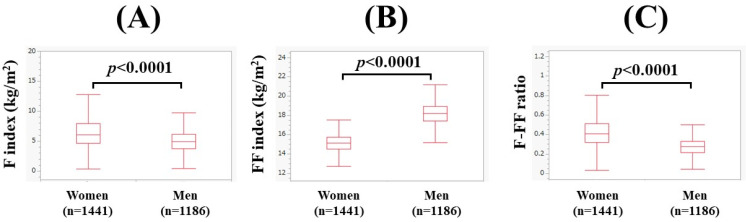
Comparison of body composition-related parameters between genders (1186 men and 1441 women). (**A**) F index, (**B**) FF index, and (**C**) F-FF ratio. The F-FF ratio was defined as the F index divided by the FF index.

The median (range) F index was 4.9 kg/m^2^ (0.45–23.2 kg/m^2^) in men and 6.1 kg/m^2^ (0.39–20.2 kg/m^2^) in women (*p* < 0.0001, Figure 1A). The median (range) FF index was 18.2 kg/m^2^ (13.4–23.7 kg/m^2^) in men and 15.1 kg/m^2^ (12.1–18.4 kg/m^2^) in women (*p* < 0.0001, Figure 1B). The median F-FF ratio was 0.272 (0.031–1.023) in men and 0.405 (0.030–1.169) in women (*p* < 0.0001, Figure 1C).

### 4.2. The Correlation between HOMA-IR and Body Composition Parameters in Men and Women

**Figure 2:**
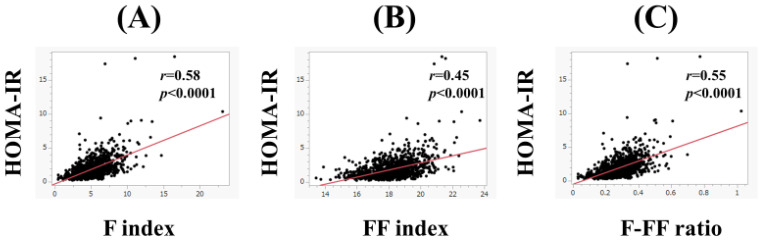
The correlation between HOMA-IR and body composition parameters in men. (**A**) F index, (**B**) FF index, and (**C**) F-FF ratio. The F-FF ratio was defined as the F index divided by the FF index.

**Figure 3:**
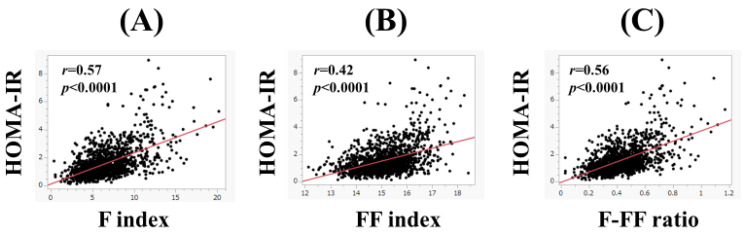
The correlation between HOMA-IR and body composition parameters in women. (**A**) F index, (**B**) FF index, and (**C**) F-FF ratio.

In men, the F index (*r* = 0.58, *p* < 0.0001), FF index (*r* = 0.45, *p* < 0.0001), and F-FF ratio (*r* = 0.55, *p* < 0.0001) were significantly correlated with HOMA-IR (Figure 2A–C). Likewise, in women, the F index (*r* = 0.57, *p* < 0.0001), FF index (*r* = 0.42, *p* < 0.0001), and F-FF ratio (*r* = 0.56, *p* < 0.0001) were significantly correlated with HOMA-IR (Figure 3A–C).

### 4.3. Subgroup Analysis 1: The Correlation between HOMA-IR and Body Composition Parameters in Men and Women According to Age

**Figure 4:**
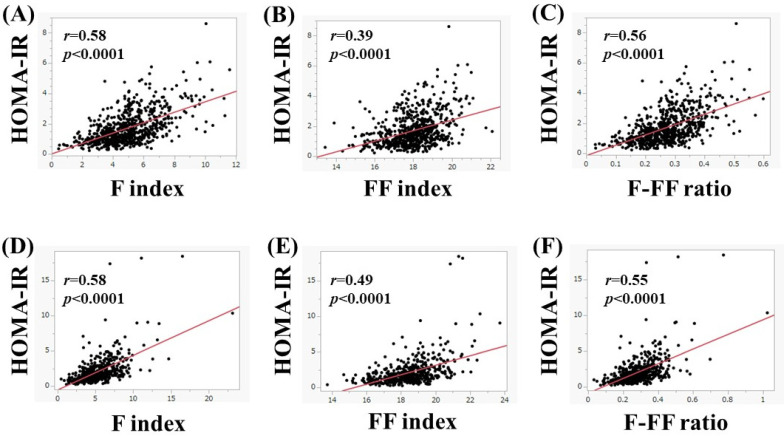
(**A**–**C**) The correlation between HOMA-IR and body composition parameters in men aged 65 years or more (*n* = 680). (**A**) F index, (**B**) FF index, and (**C**) F-FF ratio. (**D**–**F**) The correlation between HOMA-IR and body composition parameters in men aged less than 65 years (*n* = 506). (**D**) F index, (**E**) FF index, and (**F**) F-FF ratio.

In men, in subjects aged 65 years or more (*n* = 680), the F index (*r* = 0.58, *p* < 0.0001), FF index (*r* = 0.39, *p* < 0.0001), and F-FF ratio (*r* = 0.56, *p* < 0.0001) were significantly correlated with HOMA-IR (Figure 4A–C). Likewise, in subjects less than 65 years (*n* = 506), the F index (*r* = 0.58, *p* < 0.0001), FF index (*r* = 0.49, *p* < 0.0001), and F-FF ratio (*r* = 0.55, *p* < 0.0001) were significantly correlated with HOMA-IR (Figure 4D–F).

**Figure 5:**
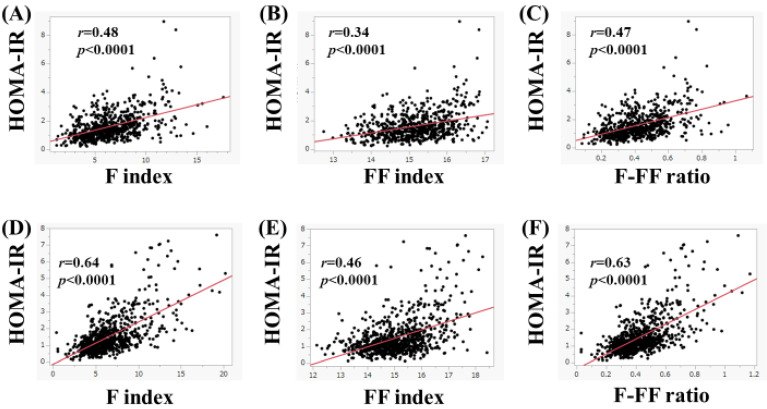
(**A**–**C**) The correlation between HOMA-IR and body composition parameters in women aged 65 years or more (*n* = 656). (**A**) F index, (**B**) FF index, and (**C**) F-FF ratio. (**D**–**F**) The correlation between HOMA-IR and body composition parameters in women aged less than 65 years (*n* = 785). (**D**) F index, (**E**) FF index, and (**F**) F-FF ratio.

In women, in subjects aged 65 years or more (*n* = 656), the F index (*r* = 0.48, *p* < 0.0001), FF index (*r* = 0.34, *p* < 0.0001), and F-FF ratio (*r* = 0.47, *p* < 0.0001) were significantly correlated with HOMA-IR (Figure 5A–C). Likewise, in subjects less than 65 years (*n* = 785), the F index (*r* = 0.64, *p* < 0.0001), FF index (*r* = 0.46, *p* < 0.0001), and F-FF ratio (*r* = 0.63, *p* < 0.0001) were significantly correlated with HOMA-IR (Figure 5D–F).

### 4.4. Subgroup Analysis 2: The Correlation between HOMA-IR and Body Composition Parameters in Men and Women According to the Presence of FL

**Figure 6:**
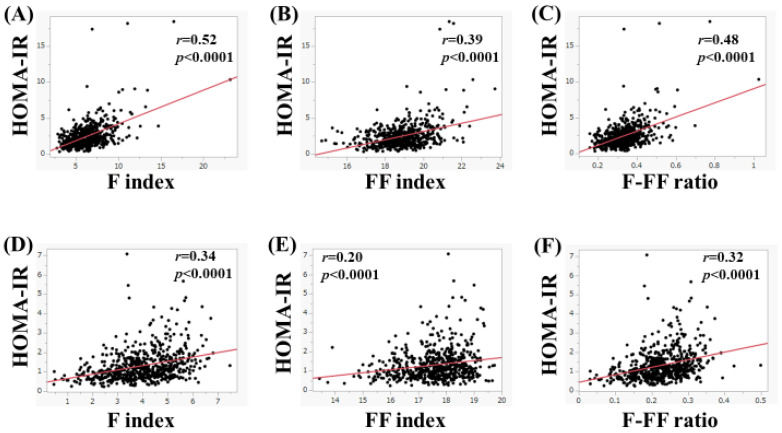
(**A**–**C**) The correlation between HOMA-IR and body composition parameters in men with fatty liver upon ultrasonography (*n* = 582). (**A**) F index, (**B**) FF index, and (**C**) F-FF ratio. (**D**–**F**) The correlation between HOMA-IR and body composition parameters in men without fatty liver on ultrasonography (*n* = 604). (**D**) F index, (**E**) FF index, and (**F**) F-FF ratio.

In men, in subjects with FL upon US (*n* = 582), the F index (*r* = 0.52, *p* < 0.0001), FF index (*r* = 0.39, *p* < 0.0001), and F-FF ratio (*r* = 0.48, *p* < 0.0001) were significantly correlated with HOMA-IR (Figure 6A–C). Likewise, in subjects without FL upon US (*n* = 604), the F index (*r* = 0.34, *p* < 0.0001), FF index (*r* = 0.20, *p* < 0.0001), and F-FF ratio (*r* = 0.32, *p* < 0.0001) were significantly correlated with HOMA-IR (Figure 6D–F).

**Figure 7:**
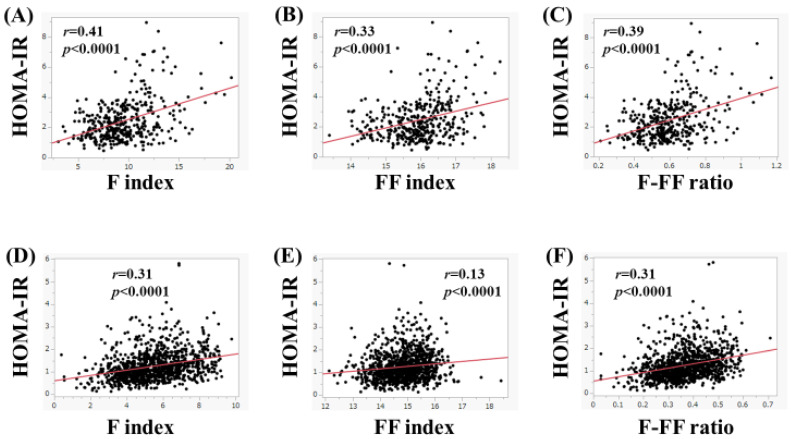
(**A**–**C**) The correlation between HOMA-IR and body composition parameters in women with fatty liver upon ultrasonography (*n* = 385). (**A**) F index, (**B**) FF index, and (**C**) F-FF ratio. (**D**–**F**) The correlation between HOMA-IR and body composition parameters in women without fatty liver on ultrasonography (*n* = 1056). (**D**) F index, (**E**) FF index, and (**F**) F-FF ratio.

In women, in subjects with FL upon US (*n* = 385), the F index (*r* = 0.41, *p* < 0.0001), FF index (*r* = 0.33, *p* < 0.0001), and F-FF ratio (*r* = 0.39, *p* < 0.0001) were significantly correlated with HOMA-IR (Figure 7A–C). Likewise, in subjects without FL upon US (*n* = 1056), the F index (*r* = 0.31, *p* < 0.0001), FF index (*r* = 0.13, *p* < 0.0001), and F-FF ratio (*r* = 0.31, *p* < 0.0001) were significantly correlated with HOMA-IR (Figure 7D–F).

### 4.5. Subgroup Analysis 3: The Correlation between HOMA-IR and Body Composition Parameters in Men and Women According to BMI

**Figure 8:**
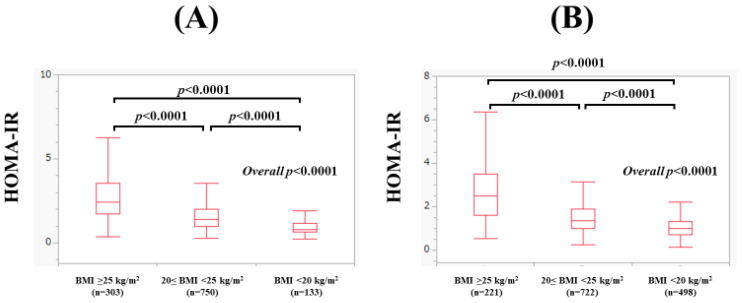
(**A**) Comparison of HOMA-IR among subjects with subjects with a BMI of 25 kg/m^2^ or more (*n* = 303), 20 kg/m^2^ ≤ BMI < 25 kg/m^2^ (*n* = 750), and BMI < 20 kg/m^2^ (*n* = 133) in men. (**B**) Comparison of HOMA-IR among subjects with subjects with a BMI of 25 kg/m^2^ or more (*n* = 221), 20 kg/m^2^ ≤ BMI < 25 kg/m^2^ (*n* = 722), and BMI < 20 kg/m^2^ (*n* = 498) in women.

**Figure 9:**
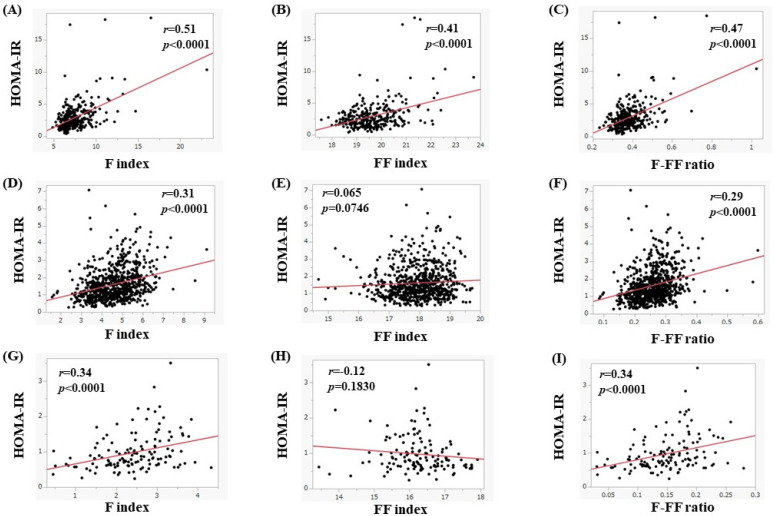
(**A**–**C**) The correlation between HOMA-IR and body composition parameters in men with a BMI of 25 kg/m^2^ or more (*n* = 303). (**A**) F index, (**B**) FF index, and (**C**) F-FF ratio. (**D**–**F**) The correlation between HOMA-IR and body composition parameters in men with a 20 ≤ BMI < 25 kg/m^2^ (*n* = 750). (**D**) F index, (**E**) FF index, and (**F**) F-FF ratio. (**G**–**I**) The correlation between HOMA-IR and body composition parameters in men with a BMI less than 20 kg/m^2^ (*n* = 133). (**G**) F index, (**H**) FF index, and (**I**) F-FF ratio.

In men, the median (range) HOMA-IR in subjects with a BMI of 25 kg/m^2^ or more (*n* = 303), 20 kg/m^2^ ≤ BMI < 25 kg/m^2^ (*n* = 750), and BMI < 20 kg/m^2^ (*n* = 133) was 2.44 (0.38–18.45), 1.39 (0.27–7.08), and 0.81 (0.24–3.50) (overall *p* < 0.0001, Figure 8A), respectively. In subjects with a BMI of 25 kg/m^2^ or more, the F index (*r* = 0.51, *p* < 0.0001), FF index (*r* = 0.41, *p* < 0.0001), and F-FF ratio (*r* = 0.47, *p* < 0.0001) were significantly correlated with HOMA-IR (Figure 9A–C). In subjects with a 20 kg/m^2^ ≤ BMI < 25 kg/m^2^ (*n* = 750), the F index (*r* = 0.31, *p* < 0.0001) and F-FF ratio (*r* = 0.29, *p* < 0.0001) were significantly correlated with HOMA-IR, while the FF index did not significantly correlate with HOMA-IR (*r* = 0.065, *p* = 0.0746) (Figure 9D–F). In subjects with a BMI < 20 kg/m^2^, the F index (*r* = 0.34, *p* < 0.0001) and F-FF ratio (*r* = 0.34, *p* < 0.0001) were significantly correlated with HOMA-IR, while the FF index did not significantly correlate with HOMA-IR (*r* = −0.12, *p* = 0.1830) (Figure 9G–I).

**Figure 10:**
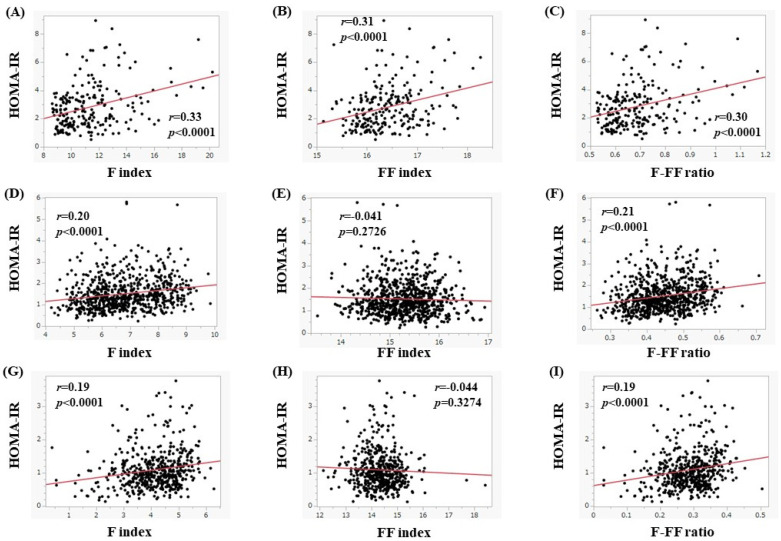
(**A**–**C**) The correlation between HOMA-IR and body composition parameters in women with a BMI of 25 kg/m^2^ or more (*n* = 221). (**A**) F index, (**B**) FF index, and (**C**) F-FF ratio. (**D**–**F**) The correlation between HOMA-IR and body composition parameters in women with a 20 ≤ BMI < 25 kg/m^2^ (*n* = 722). (**D**) F index, (**E**) FF index, and (**F**) F-FF ratio. (**G**–**I**) The correlation between HOMA-IR and body composition parameters in women with a BMI less than 20 kg/m^2^ (*n* = 498). (**G**) F index, (**H**) FF index, and (**I**) F-FF ratio.

In women, the median (range) HOMA-IR in subjects with a BMI of 25 kg/m^2^ or more (*n* = 221), 20 kg/m^2^ ≤ BMI < 25 kg/m^2^ (*n* = 722), and BMI < 20 kg/m^2^ (*n* = 498) was 2.49 (0.52–8.95), 1.37 (0.24–5.81), and 0.98 (0.13–3.77) (overall *p* < 0.0001, Figure 8B), respectively. In subjects with a BMI of 25 kg/m^2^ or more, the F index (*r* = 0.33, *p* < 0.0001), FF index (*r* = 0.31, *p* < 0.0001), and F-FF ratio (*r* = 0.30, *p* < 0.0001) were significantly correlated with HOMA-IR (Figure 10A–C). In subjects with a 20 kg/m^2^ ≤ BMI < 25 kg/m^2^, the F index (*r* = 0.20, *p* < 0.0001) and F-FF ratio (*r* = 0.21, *p* < 0.0001) were significantly correlated with HOMA-IR, whereas the FF index did not significantly correlate with HOMA-IR (*r* = −0.041, *p* = 0.2726) (Figure 10D–F). In subjects with a BMI < 20 kg/m^2^, the F index (*r* = 0.19, *p* < 0.0001) and F-FF ratio (*r* = 0.19, *p* < 0.0001) were significantly correlated with HOMA-IR, while the FF index did not significantly correlate with HOMA-IR (*r* = −0.044, *p* = 0.3274) (Figure 10G–I).

### 4.6. The Correlation between the F Index and FF Index

**Figure 11:**
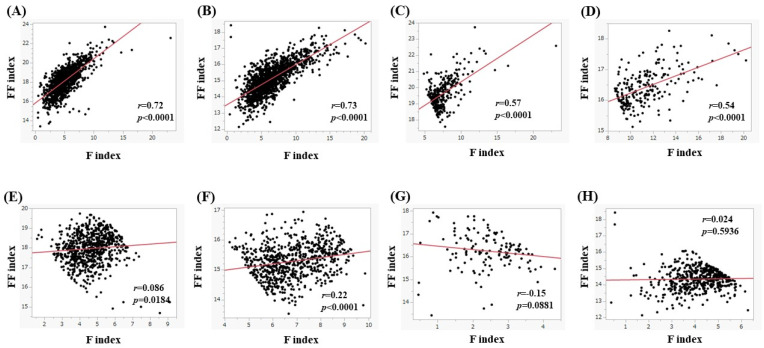
(**A**,**B**) The correlation between the F index and FF index in men (**A**) and women (**B**) for all cases. (**C**,**D**) The correlation between the F index and FF index in men (**C**) and women (**D**) in subjects with a BMI of 25 kg/m^2^ or more. (**E**,**F**) The correlation between the F index and FF index in men (**E**) and women (**F**) in subjects with a 20 kg/m^2^ ≤ BMI < 25 kg/m^2^. (**G**,**H**) The correlation between the F index and FF index in men (**G**) and women (**H**) in subjects with a BMI < 20 kg/m^2^.

The correlation between the F index and the FF index is shown in Figure 11. For all cases, the FF index significantly correlated with the F index both in men (*r* = 0.72, *p* < 0.0001, Figure 11A) and women (*r* = 0.73, *p* < 0.0001, Figure 11B). In subjects with a BMI of 25 kg/m^2^ or more, the FF index significantly correlated with the F index both in men (*r* = 0.57, *p* < 0.0001, Figure 11C) and women (*r* = 0.54, *p* < 0.0001, Figure 11D). In subjects with a 20 kg/m^2^ ≤ BMI < 25 kg/m^2^ or more, the FF index significantly correlated with the F index both in men (*r* = 0.086, *p* = 0.0186, Figure 11E) and women (*r* = 0.22, *p* < 0.0001, Figure 11F). In subjects with a BMI < 20 kg/m^2^, the FF index did not significantly correlate with the F index in both men (*r* = −0.15, *p* = 0.0881, Figure 11G) and women (*r* = 0.024, *p* = 0.5936, Figure 11H).

### 4.7. Univariate and Multivariate Analyses of Factors Linked to HOMA-IR

In men, age, BMI, systolic blood pressure, diastolic blood pressure, fasting blood sugar (FBS), platelet count, serum albumin, aspartate aminotransferase (AST), alanine aminotransferase (ALT), alkaline phosphatase (ALP), gamma-glutamyl transferase (GGT), uric acid, triglycerides, F index, FF index, and F-FF ratio were significant factors correlated with HOMA-IR (Table 2). These sixteen factors were subsequently entered into a multivariate regression analysis. In the multivariate analysis, serum albumin (*p =* 0.020), FBS (*p* < 0.0001), ALT (*p* < 0.0001), GGT (*p* = 0.0191), triglycerides (*p* < 0.0001), and F-FF ratio (*p* < 0.0001) were found to be significant factors associated with HOMA-IR (Table 2).

In women, BMI, systolic blood pressure, diastolic blood pressure, FBS, platelet count, serum albumin, AST, ALT, ALP, GGT, uric acid, total cholesterol, triglycerides, F index, FF index, and F-FF ratio were significant factors correlated with HOMA-IR (Table 3). These sixteen factors were subsequently entered into a multivariate regression analysis. In the multivariate analysis, serum albumin (*p* < 0.0001), FBS (*p* < 0.0001), AST (*p* = 0.0164), ALT (*p* < 0.0001), total cholesterol (*p* < 0.0001), triglycerides (*p* < 0.0001), and F-FF ratio (*p* < 0.0001) were found to be significant factors associated with HOMA-IR (Table 3).

## 5. Discussion

Obesity is only an accumulation of adipose cells and does not simply refer to a heavy body weight [16]. Obesity is also indicated by an imbalance in energy, with energy intake exceeding energy expenditure and excessive accumulation of body fat [17]. Currently, BMI is primarily used as the evaluation method for nutritional assessment; however, given that body composition is classified into the three components of body fat, bone, and lean tissue, BMI alone is not sufficient for evaluation. On the other hand, obesity is closely linked to IR. In this study, we sought to examine the relationship between HOMA-IR and accurate body composition values by BIA. The contribution of research on body composition to the development of anatomy is significant. As far as we are aware, this study is one of the largest Japanese studies regarding the relationship between IR and body-composition-related parameters in Japanese health check-up recipients.

In the summary of our results, the F-FF ratio was an independent factor associated with HOMA-IR in the multivariate analysis of both genders, while the F index and FF index were not in both genders. As previously reported, factors associated with protein and lipid metabolism also became independent factors related to HOMA-IR in our analysis [18]. Moreover, in all subgroup analyses, the F-FF ratio significantly correlated with HOMA-IR in both genders. Our results denoted that the F-FF ratio can be a predictor of IR in both genders in Japanese adults. Seo YG, et al. reported that a higher fat-to-muscle ratio (i.e., F-FF ratio) was significantly associated with the prevalence of IR, which is in line with our results [11]. The fat-to-muscle ratio was also reported to be associated with coronary artery disease in healthy adults [19], the development or deterioration of nonalcoholic fatty liver disease (NAFLD) [20], metabolic syndrome [21], muscle strength [22], poor clinical outcome in COVID-19 disease [23], risk of dementia [24], decreased renal function [25], and all-cause mortality [26,27]. Researchers are thus becoming increasingly interested in the balance between fat and skeletal muscle in the human body. This seems to be an important point of view considering the organ-organ network.

In our results, body composition parameters significantly differed between genders. Notably, although BMI in women was significantly lower than that in men, the F index in women was significantly higher than that in men. BMI alone should not be used to estimate body fat mass. While FL upon US was found in 582 subjects (49.1%) in men and 385 subjects (26.7%) in women (overall: 36.8% (967/2627)), Ito T, et al. reported in their meta-analysis of Japanese studies published between 1984 and 2012 that the overall NAFLD prevalence was 25.5% (the prevalence was higher in men, varied by region, and increased over time), which was considerably lower than our data [28]. Our present results were based on data between February 2022 and May 2023. In view of this, the NAFLD prevalence in our country seems to be steadily increasing.

One possible reason why the F-FF ratio was extracted as an independent factor in multivariate analysis is the involvement of inflammatory cytokines and adipokines. In the obese state, chronic inflammation characterized by cellular infiltration of macrophages and other cells into adipose and liver tissue occurs [29]. Such chronic inflammation induces IR by altering adipokine secretion and inducing oxidative and endoplasmic reticulum stress [30]. Adipose tissue in obese subjects, especially visceral adipose tissue, is infiltrated with numerous macrophages (M1 macrophages [31]), which secrete abundant proinflammatory cytokines and adipokines, including tumor necrosis factor-α. These inflammatory cytokines and adipokines act on skeletal muscle and the liver to induce IR [32]. The involvement of inflammatory cytokines in the adipose-skeletal muscle interaction is closely related to the results of this study (among the F index, FF index, and F-FF ratio, only the F-FF ratio was an independent factor). On the other hand, as shown in Figure 9 and Figure 10, the correlation coefficients between the F index and F-FF ratio and HOMA-IR tended to be lower for both men and women with a BMI below 25 kg/m^2^ than for those with a BMI above 25 kg/m^2^. This is probably due to the fact that activation of the inflammatory cytokines or adipokines is attenuated in non-obese individuals [33,34,35].

The correlation coefficients between the F index and FF index were shown in the present study to differ markedly by BMI, with the correlation coefficients decreasing with decreasing BMI in both men and women. Despite this, it is interesting to note that HOMA-IR was significantly correlated with the F-FF ratio in both men and women, even when examined by BMI. IR is thought to robustly regulate the fat-skeletal muscle balance. Twenty percent of Japanese NAFLD patients have NAFLD without obesity (i.e., lean NAFLD) [28], and the involvement of the patatin-like phospholipase domain-containing 3 protein (PNPLA3) gene has been reported [36]. In NAFLD with obesity, improvement can be expected with appropriate dietary and exercise interventions [37]. Although appropriate intervention is difficult in lean NAFLD [37], it is possible that maintaining an appropriate body composition balance using HOMA-IR as an indicator may improve lean NAFLD.

We must acknowledge several limitations of the current study. First, this study was a single-center cross-sectional observational study with a retrospective analysis, which was limited to Japanese adults. Second, all medication was based on self-report, potentially creating bias. Thus, data should be carefully interpreted. Nevertheless, our data indicated that neither the F index alone nor the FF index alone but the F-FF index was closely linked to HOMA-IR. As a drug discovery project for IR, we would like to consider drug development with inter-organ networks in mind. In addition, balanced dietary intervention may be effective in ameliorating metabolic indices, including IR [38].

## 6. Conclusions

In conclusion, fat and skeletal muscle balance can be controlled by IR in Japanese adults.

## Figures and Tables

**Table 1:** Baseline characteristics.

	Men (*n* = 1186)	Women (*n* = 1441)	*p* Value
Age (years)	67 (25–89)	63 (20–90)	<0.0001
Body mass index (kg/m^2^)	23.0 (14.3–45.7)	21.2 (13.3–37.5)	<0.0001
Systolic blood pressure (mmHg)	125 (80–190)	120 (78–192)	<0.0001
Diastolic blood pressure (mmHg)	79 (45–121)	73 (38–110)	<0.0001
Fatty liver on ultrasonography, yes/no	582/604	385/1056	<0.0001
HbA1c (%)	5.7 (4.8–10.8)	5.7 (4.6–10.9)	0.1100
HOMA-IR	1.54 (0.24–18.45)	1.30 (0.13–8.95)	<0.0001
Fasting blood sugar (mg/dL)	93 (67–298)	89 (63–111)	<0.0001
Platelet count (×10^4^/μL)	22.6 (7.2–64.5)	24.3 (7.8–51.7)	<0.0001
Serum albumin (g/dL)	4.3 (3.4–5.1)	4.3 (3.3–5.3)	0.1101
AST (IU/L)	22 (11–115)	21 (10–87)	<0.0001
ALT (IU/L)	20 (6–181)	16 (5–146)	<0.0001
ALP (IU/L)	67 (24–187)	65 (19–218)	0.1587
GGT (IU/L)	27 (8–314)	18 (3–209)	<0.0001
eGFR (mL/min/1.73 m^2^)	65.9 (29.4–110.8)	68.8 (23.9–139.7)	<0.0001
Uric acid (mg/dL)	6.1 (2.2–10.4)	4.8 (0.7–9.4)	<0.0001
Total cholesterol (mg/dL)	206 (90–326)	220 (129–356)	<0.0001
Triglyceride (mg/dL)	93.5 (33–826)	76 (20–360)	<0.0001
Habitual smoking, yes/no	194/992	60/1381	<0.0001
Habitual drinking, yes/no	500/686	270/1171	<0.0001
Fat mass index (kg/m^2^)	4.9 (0.45–23.2)	6.1 (0.39–20.2)	<0.0001
Free fat mass index (kg/m^2^)	18.2 (13.4–23.7)	15.1 (12.1–18.4)	<0.0001
F-FF ratio	0.272 (0.031–1.023)	0.405 (0.030–1.169)	<0.0001

Data are shown as numbers or median (range). HOMA-IR; homeostasis model assessment of insulin resistance, AST; aspartate aminotransferase, ALT; alanine aminotransferase, ALP; alkaline phosphatase, GGT: γ-glutamyl transpeptidase, eGFR; estimated glomerular filtration rate, F index: fat mass index, FF index: fat-free mass index, F-FF ratio was defined as F index divided by FF index.

**Table 2:** Univariate and multivariate analyses of factors linked to HOMA-IR in men.

**(A)**			
**Men**		***r***	***p* Value**
Age		−0.098	0.0008
Body mass index		0.57	<0.0001
Systolic blood pressure		0.18	<0.0001
Diastolic blood pressure		0.22	<0.0001
Fasting blood sugar		0.35	<0.0001
Platelet count		0.16	<0.0001
Serum albumin		0.14	<0.0001
AST		0.25	<0.0001
ALT		0.43	<0.0001
ALP		0.14	<0.0001
GGT		0.22	<0.0001
eGFR		−0.049	0.0899
Uric acid		0.15	<0.0001
Total cholesterol		0.045	0.1228
Triglyceride		0.38	<0.0001
F index		0.58	<0.0001
FF index		0.45	<0.0001
F-FF ratio		0.55	<0.0001
**(B)**			
**Multivariate**	**Estimates**	**Standard Error**	***p* Value**
Age	0.0013	0.0030	0.6623
Body mass index	0.102	1.020	0.9203
Systolic blood pressure	0.00052	0.0028	0.8527
Diastolic blood pressure	0.0018	0.0039	0.6520
Fasting blood sugar	0.0273	0.0023	<0.0001
Platelet count	0.0099	0.0056	0.0793
Serum albumin	0.2885	0.1238	0.020
AST	−0.0094	0.0061	0.1230
ALT	0.0177	0.0038	<0.0001
ALP	0.0027	0.0017	0.1022
GGT	−0.0024	0.0010	0.0191
Uric acid	0.0106	0.0260	0.6847
Triglyceride	0.0043	0.0005	<0.0001
F index	1.474	1.030	0.1537
FF index	−0.418	1.022	0.6827
F-FF ratio	−24.109	3.084	<0.0001

HOMA-IR; homeostasis model assessment of insulin resistance, AST; aspartate aminotransferase, ALT; alanine aminotransferase, ALP; alkaline phosphatase, GGT: γ-glutamyl transpeptidase, eGFR; estimated glomerular filtration rate, F index: fat mass index, FF index: fat free mass index, F-FF ratio was defined as F index divided by FF index.

**Table 3:** Univariate and multivariate analyses of factors linked to HOMA-IR in women.

**(A)**			
**Women**		***r***	***p* Value**
Age		0.038	0.1542
Body mass index		0.56	<0.0001
Systolic blood pressure		0.25	<0.0001
Diastolic blood pressure		0.21	<0.0001
Fasting blood sugar		0.44	<0.0001
Platelet count		0.15	<0.0001
Serum albumin		0.056	0.0344
AST		0.17	<0.0001
ALT		0.37	<0.0001
ALP		0.20	<0.0001
GGT		0.25	<0.0001
eGFR		0.012	0.6418
Uric acid		0.27	<0.0001
Total cholesterol		−0.065	0.0131
Triglyceride		0.36	<0.0001
F index		0.57	<0.0001
FF index		0.42	<0.0001
F-FF ratio		0.56	<0.0001
**(B)**			
**Multivariate**	**Estimates**	**Standard Error**	***p* Value**
Body mass index	0.6792	0.6904	0.3254
Systolic blood pressure	0.0018	0.0018	0.3255
Diastolic blood pressure	0.00034	0.0027	0.8998
Fasting blood sugar	0.0264	0.0020	<0.0001
Platelet count	0.0027	0.0037	0.4649
Serum albumin	0.3819	0.0852	<0.0001
AST	−0.0136	0.0056	0.0164
ALT	0.0222	0.0036	<0.0001
ALP	0.0021	0.0011	0.0562
GGT	−0.00024	0.0013	0.8560
Uric acid	0.0031	0.0223	0.8911
Total cholesterol	−0.0034	0.00062	<0.0001
Triglyceride	0.0032	0.00052	<0.0001
F index	0.1557	0.6975	0.8234
FF index	−0.9230	0.6937	0.1836
F-FF ratio	−10.9951	1.8862	<0.0001

HOMA-IR; homeostasis model assessment of insulin resistance, AST; aspartate aminotransferase, ALT; alanine aminotransferase, ALP; alkaline phosphatase, GGT: γ-glutamyl transpeptidase, eGFR; estimated glomerular filtration rate, F index: fat mass index, FF index: fat free mass index, F-FF ratio was defined as F index divided by FF index.

## Data Availability

Data available on request due to restrictions, e.g., privacy or ethical.

## Abbreviations

IR; insulin resistance, BIA; bioelectrical impedance analysis, HOMA-IR; Homeostasis Model Assessment of Insulin Resistance, OMPU; Osaka Medical and Pharmaceutical University, F index; fat mass index, FF index; fat free mass index, F-FF ratio; F index to FF index ratio, BMI; body mass index, FL; fatty liver, US; ultrasonography, FBS; fasting blood sugar, AST; aspartate aminotransferase, ALT; alanine aminotransferase, ALP; alkaline phosphatase, GGT; gamma-glutamyl transferase, NAFLD; nonalcoholic fatty liver disease
